# High methionine diet mediated oxidative stress and proteasome impairment causes toxicity in liver

**DOI:** 10.1038/s41598-024-55857-1

**Published:** 2024-03-06

**Authors:** Faouzia Derouiche, Randa Djemil, Fatima Zohra Sebihi, Lilia Douaouya, Hichem Maamar, Katia Benjemana

**Affiliations:** 1 Biotechnology, Water, Environment and Health Laboratory, Faculty of Natural and Life Sciences, University Abbes Lagherour, Khenchela, Algeria; 2 Department of Molecular and Cellular Biology, Faculty of Natural and Life Sciences, University Abbes Lagherour, Khenchela, Algeria

**Keywords:** Biochemistry, Cell biology, Biomarkers, Diseases

## Abstract

Methionine (Met) rich diet inducing oxidative stress is reported to alter many organs. Proteasome as a regulator of oxidative stress can be targeted. This study was performed to investigate if excessive methionine supplementation causes hepatotoxicity related to proteasome dysfunction under endogenous oxidative stress in rats. Male Wistar albino rats (n = 16) were divided into controls and treated groups. The treated rats (n = 08) received orally l-methionine (1 g/kg/day) for 21 days. Total homocysteine (tHcy), total oxidant status (TOS), total antioxidant status (TAS), hepatic enzymes levels: aspartate aminotransferase (AST), alanine aminotransferase (ALT), lactate dehydrogenase (LDH), alkaline phosphatase (ALP), with total bilirubin (TBil), albumin (Alb), and C-reactive protein (CRP) were determined in plasma by biochemical assays. Liver supernatants were used for malondialdehyde (MDA), protein carbonyls (PC), glutathione (GSH), catalase (CAT), superoxide dismutase (SOD), glutathione peroxidase (GPx), 20S proteasome activities and their subunits expression, tumor necrosis factor-α (TNF-α), and interleukin 6 (IL-6) evaluation by appropriate methods and light microscopy for liver histological examination. Methionine treatment increased homocysteine, TOS, oxidative stress index (OSI), MDA and PC but decreased TAS, GSH, CAT, SOD, GPx with the 20S proteasome activities and their β subunits expression. Liver proteins: AST, ALT, LDH, ALP, TBil and CRP were increased but Alb was decreased. Liver histology was also altered. An increase in liver TNF-α and IL-6 levels were observed. These findings indicated that methionine supplementation associated oxidative stress and proteasome dysfunction, caused hepatotoxicity and inflammation in rat. Further investigations should be to better understand the relation between methionine, oxidative stress, proteasome, and liver injuries.

## Introduction

Methionine (Met) is an essential sulfur-containing amino acid obtained from food in humans. Despite, methionine is mainly metabolized in liver and acts as antioxidant, being the precursor for glutathione (GSH) synthesis, and also the principal methyl donor for important biological reactions such as DNA methylation and protein synthesis, when it is consumed in excess, caused pathophysiological effects on the liver including cell injuries and dysfunction^1^, throughout hyperhomocysteinemia (HHcy) or elevated homocysteine (Hcy) that mediated cellular oxidative stress by generation of intracellular reactive oxygen species (ROS)^2^ via homocysteine autooxidation. Methionine is converted to homocysteine, a non-proteinogenic aminothiol metabolite by successive enzymatic reactions^1,3^. Three categories of homocysteine levels, normal (5–15 μmol/l), moderate (15–30 μmol/l), intermediate (30–100 μmol/l) and severe (> 100 μmol/l), related to genetic defects of Met/Hcy metabolism, drugs, diseases, vitamins B and folate deficiencies^2^.

Numerous studies associated hyperhomocysteinemia to cardiovascular, kidney, diabetes, neurodegenerative, some types of cancer and other diseases^4–9^ by causing multi-organs damages and inflammation during oxidative stress^10,11^. Among multiple liver diseases including hepatitis C, autoimmune hepatitis, hemochromatosis, alcohol-fatty liver disease, non-alcohol fatty liver disease (NAFLD) and hepatocarcinoma, caused by viral infections, immune, genetic, metabolic conditions and cancer, it reported that high methionine induced hyperhomocysteinemia associated hepatotoxicity in NAFLD liver diseases^1,12,13^.

Oxidative stress is an imbalance between increased prooxidants and insufficient antioxidants that occurs when excessive production of reactive oxygen and nitrogen species (ROS/RNS) such as hydroxyl radical (HO^⋅^), superoxide anion (O_2_^⋅−^), hydrogen peroxide (H_2_O_2_), nitric oxide (NO^⋅^), nitroxyl anion (NO^−^), and peroxynitrite (NOOO^⋅^). Oxidative stress oxidized lipids, proteins, DNA and damaged cell functions and thus it is implicated in the pathogenesis of various diseases^14^.

Ubiquitin–proteasome system plays a crucial role in many cellular processes by regulated degradation of intracellular ubiquitinated proteins^15^. It recognizes and selectively removes oxidatively damaged proteins in response to oxidative stress^16^ and also it is involved in the control of inflammatory processes^17^. Previous studies have shown proteasome inhibition during oxidative stress^18^. Studie**s** showed proteasome dysfunction in NAFLD^19^ and alcohol liver disease (ALD)^20^. However hepatic proteasome function remains poorly documented in methionine associated hepatotoxicity.

Proteasome is an ATP- dependent multi-proteolytic complex, composed of a central catalytic core particle (CP) or 20S proteasome capped by one or two terminal 19S regulatory particle (s) (RP) or activators to form respectively, 26S proteasome or 30S proteasome. 28 subunits, arranged in two both similar outer *α*-rings and inner *β*-rings, consist the 20 CP, in side β*5,* β*2,* β*1* subunits which are respectively associated with chymotrypsin-like trypsin-like and caspase-like activities^21^.

The aim of this study is, first to create a rat model of hyperhomocysteinemia by methionine supplementation, second to investigate the impact of this model on biomarkers of oxidative stress and proteasome function in liver and finally to verify the parameters of hepatotoxicity and inflammation.

## Results

### Homocysteine and oxidative stress biomarkers

**Figure 1:**
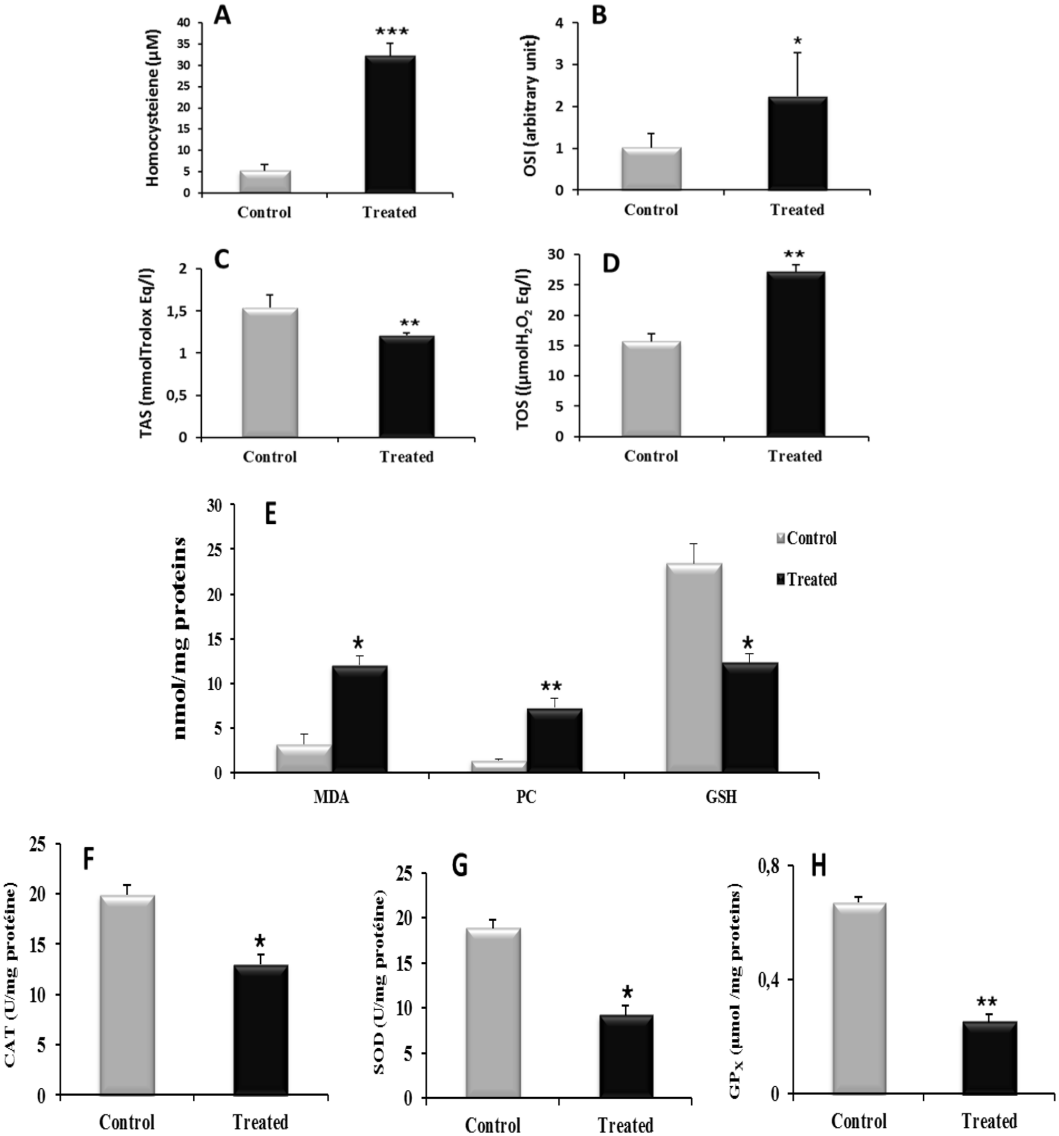
Effect of l-methionine supplementation on levels of Homocysteine (**A**), OSI (**B**), TAS (**C**), TOS (**D**), (MDA, PC,GSH) (**E**), CAT (**F**), SOD (**G**), GPx (**H**) in plasma of controls and treated rats (n = 08/group). Values were expressed as means ± SEM, (*p < 0.05, **p < 0.01, ***p < 0.001) vs controls. *MDA* malondialdehyde, *PC* protein carbonyl, *GSH* Reduced glutathione, *CAT* catalase, *SOD* Superoxide dismutase, *GPx* glutathione peroxidase.

Total homocysteine was significantly increased in l-methionine treated rats compared to controls with (6.16 fold change) (Fig. 1A).

TOS level was significantly increased and OSI was higher by (2.2 fold) (Fig. 1D–B) but TAS (Fig. 1C) decreased in treated group compared to controls.

Levels of both liver lipid peroxidation (MDA) and protein oxidation (PC) were significantly higher respectively (3.74, and 5.16 fold change) in l-methionine treated rats than in controls (Fig. 1E). On the other hand, GSH (Fig. 1E), CAT (Fig. 1F), SOD (Fig. 1G) and GPx (Fig. 1H) levels were significantly reduced in treated rats.

### Proteasome function and β subunits expression levels

**Figure 2:**
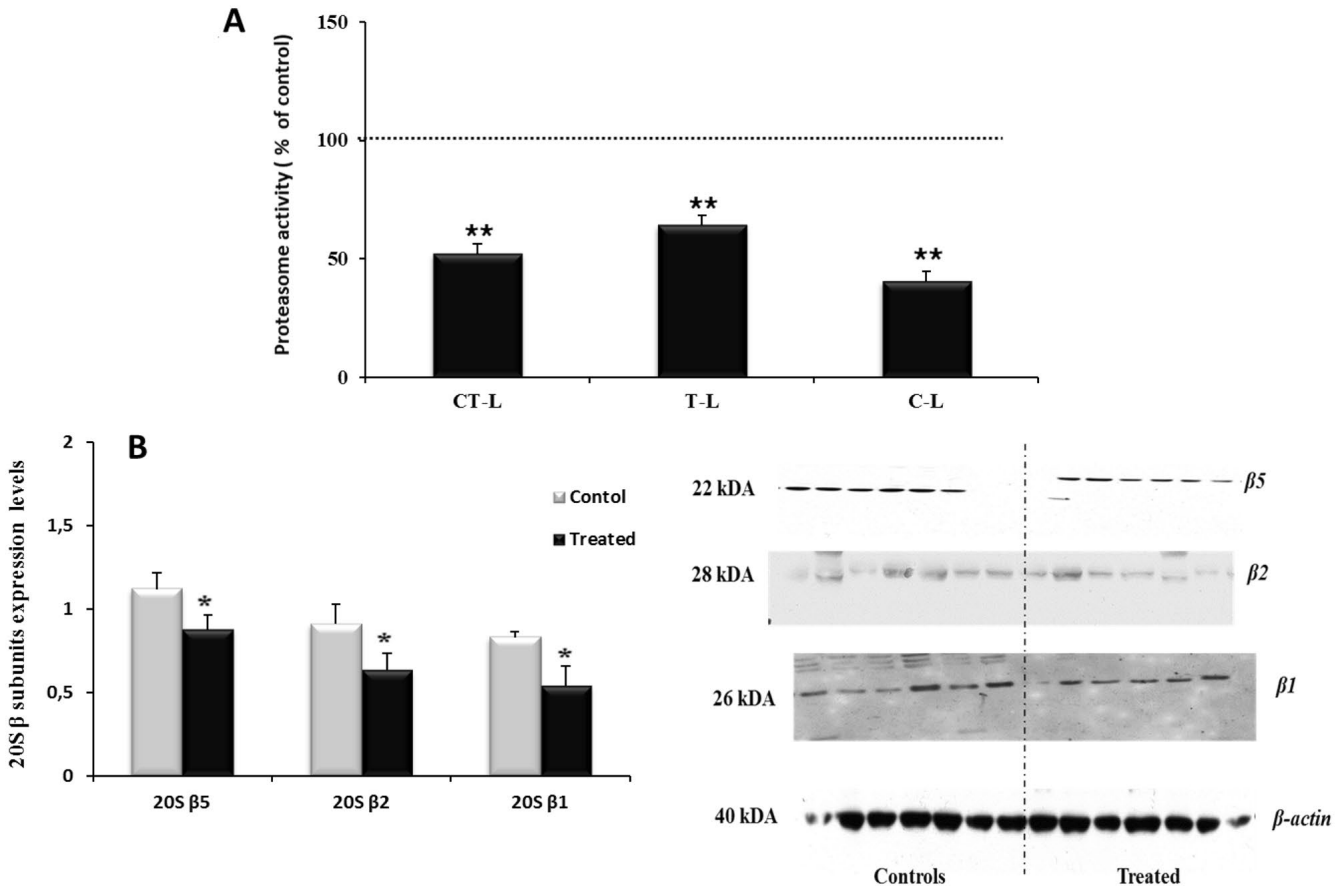
Effect of L-methionine supplementation on 20S proteasome activities (**A**) and 20S β subunits expression levels normalized to β actin (**B**) in liver of controls and treated rats, A (n = 08/group) and B (n = 06/group). Proteins were quantified by densimetry using Image scanner III and Image Quant TL software (GE Healthcare). Blots images were cutting from the films scanned to eliminate other blots appeared in full length membranes, with membrane edges. Values were expressed as means ± SEM, (*p < 0.05, **p < 0.01) vs controls. *CT-L* chymotrypsin-like, *T-L* trypsin-like, *C-L* caspase-like.

All liver 20S proteasome activities: chymotrypsin-like, trypsin-like and caspase-like were significantly decreased in treated rats compared with controls as shown in Fig. 2A. 20S β subunits (β5, β2, β1) expression levels were also significantly decreased in treated rats as compared to controls (Fig. 2B).

### Hepatic toxicity

**Figure 3:**
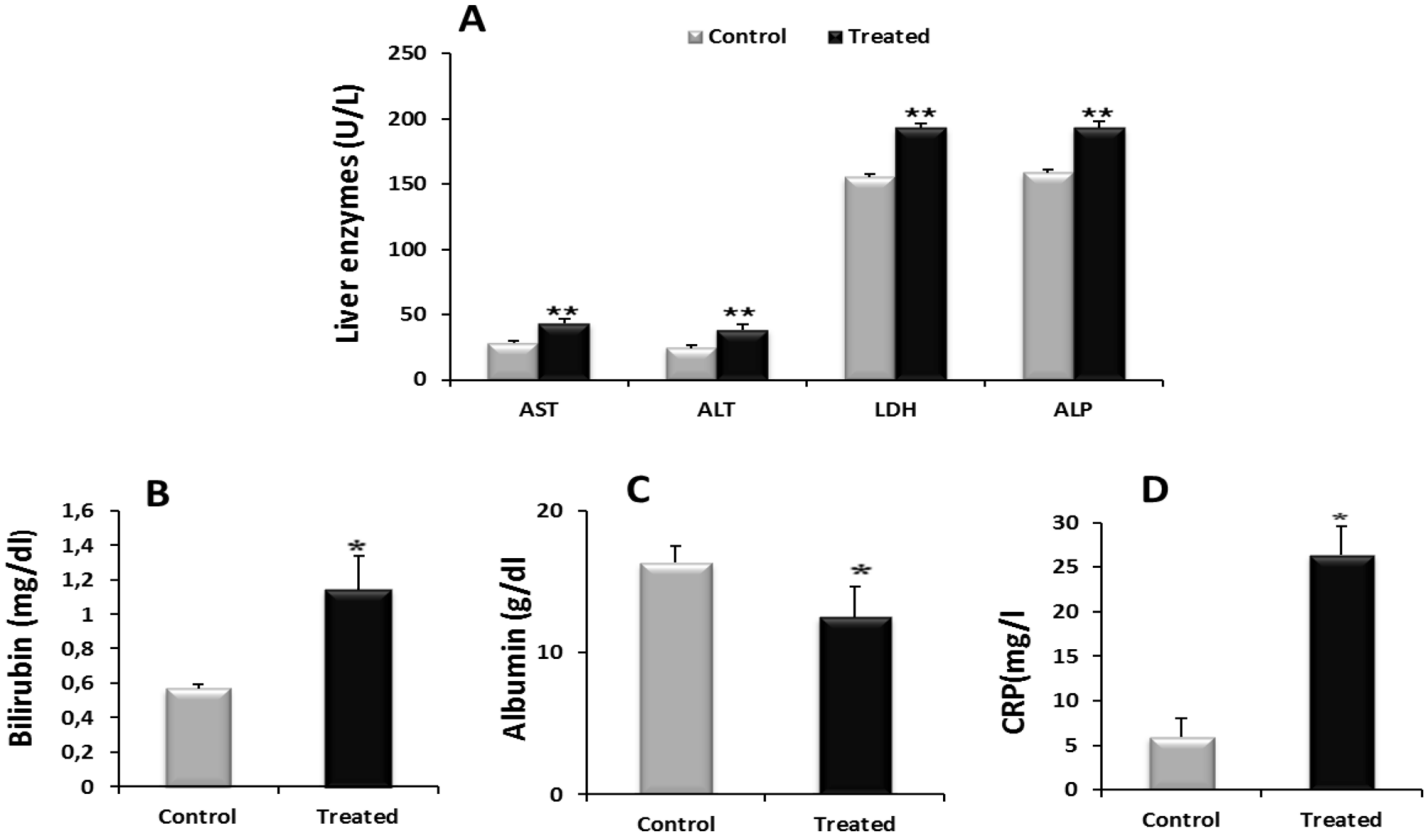
Effect of l-methionine supplementation on levels of liver biomarkers (AST, ALT, LDH, ALP) (**A**), TBil (**B**), Alb (**C**) and CRP (**D**) in plasma of controls and treated rats (n = 08/group). Values were expressed as means ± SEM, (*p < 0.05, **p < 0.01) vs controls. (*AST)* aspartate amino transferase, (*ALT)* alanine amino transferase, (*LDH)* lactate dehydrogenase, (*ALP)* alkaline phosphatase, (*TBil)* total bilirubin, (*Alb) albumin: (CRP)* C-reactive protein.

Liver proteins demonstrated a significant (p < 0.05) increase of their level in plasma (AST, ALT, LDH, ALP, TBil), Fig. 3A,B but significant decrease in the level of albumin (Alb) (Fig. 3C) in treated as compared to control rats.

### Hepatic structure

**Figure 4:**
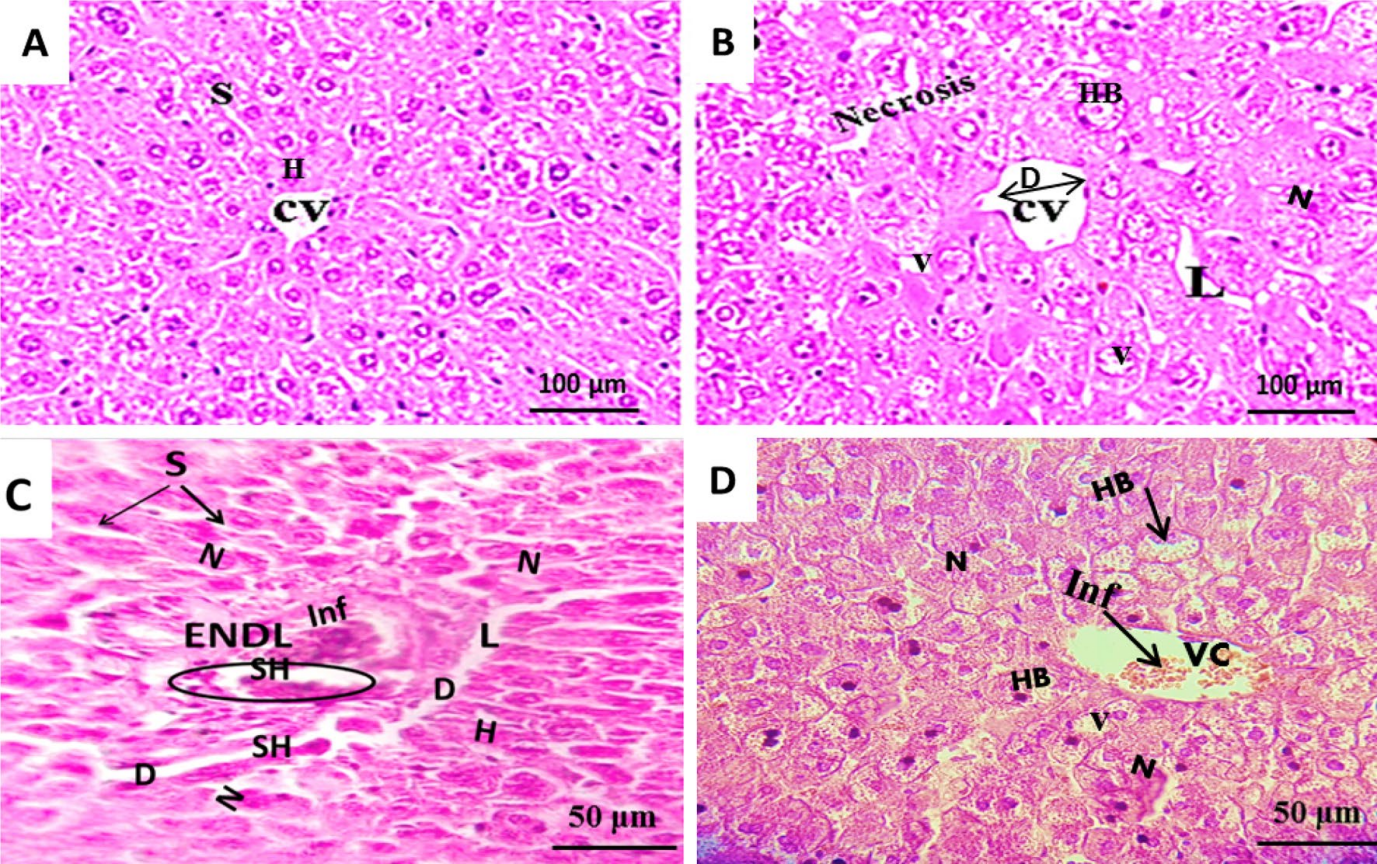
Photomicrographs of H&E (X400 magnifications) stained rat liver longitudinal sections (4 slides/animal), from controls (n = 08) (**A**) (scale bar, 100 µm) and treated rats (n = 08) with 3 weeks L-methionine (**B**) (scale bar, 100 µm) and (**C**, **D**) (scale bar, 50 µm). *H* hepatocyte, *S* sinusoid, *CV* central vein, *D* dilatation, *N* necrosis, *HB* ballooned hepatocyte, *V* vacuoles, *SH* sinusoid hemorrhage, *L* lysis, *ENDL* endolysis, *Inf* Inflammatory cells infiltration.

Microscopic investigation of the liver in control rats showed normal hepatocytes around the central vein with preserved cytoplasm (Fig. 4A). In treated rats, liver revealed significant altered hepatocytes with vacuolated cytoplasm, some areas of necrosis and inflammatory cells infiltration, also dilation of the central vein, lysis and hemorrhage at sinusoid level, ballooned hepathocytes and vacuolization (Fig. 4B–C- D).

### Hepatic inflammation

**Figure 5:**
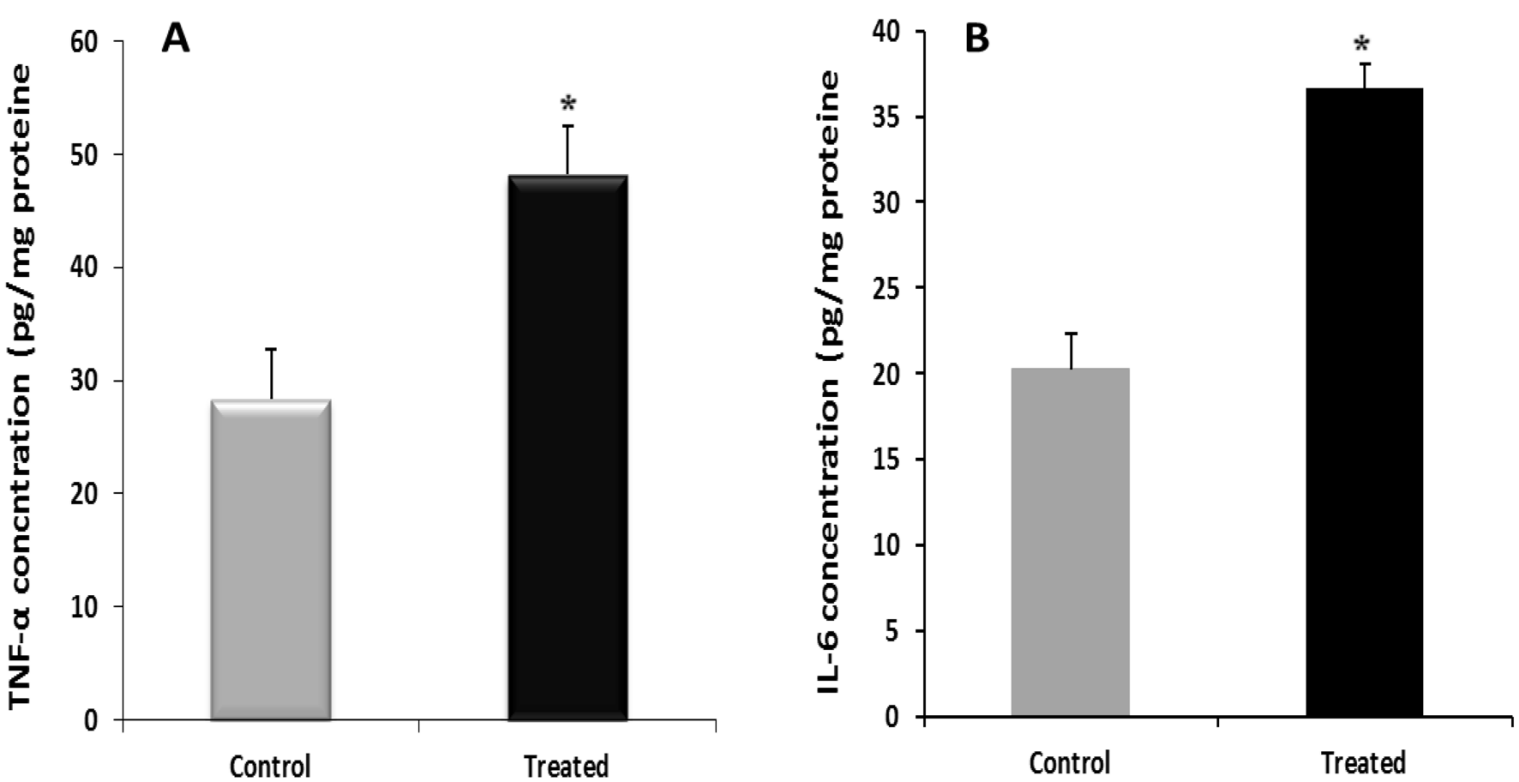
Effect of l-methionine supplementation on levels of TNF-α (**A**) and IL-6 (**B**) in liver of controls and treated rats (n = 08/group). Values were expressed as means ± SEM, *p < 0.05 Vs controls. *TNF-α* tumor necrosis factor-α*,* *IL-6* interleukin 6.

C-reactive protein (CRP) (Fig. 3D) in plasma and liver Proinflammatory cytokines (TNF-α and IL-6) levels were significantly higher in treated rats compared to controls as observed (Fig. 5A,B).

## Discussion

Liver is the main organ that performs many critical functions including, metabolism, protein synthesis, detoxification, vitamins and minerals storage, filtration and immunity. Thus, any disturbance of these liver functions may conduct to damages. It has been postulated that intake of high level dietary methionine is an important risk factor to be considered in liver dysfunction^1,22^.

In our study, high methionine diet accumulated homocysteine (Hcy) (32.15 μmol/l) at intermediate level^2^. We created a hyperhomocysteine rat model in accordance to previous studies reported that methionine supplementation produced hyperhomocysteinemia in animal models and human^1,7,12^.

Endogenous oxidative stress was induced by methionine treatment as showed by higher oxidative stress index (OSI), ratio of the increased TOS (total oxidant status) to the decreased TAC (total antioxidant status) and also by inhibited enzymatic (CAT, SOD, GPx) and non-enzymatic (GSH) antioxidants defenses. Herein, decreased antioxidants strongly explained increased OSI. Furthermore, liver MDA and P C, respectively as biomarkers of lipid and proteins oxidation were also increased. These findings are in agreement with others^1,2,23–25^, where it was reported that high methionine increased homocysteine to pathological range that enhanced generation of intracellular reactive oxygen species (ROS) via Hcy autooxidation and altered antioxidants. Excessive production of ROS interacted with cellular membrane and components leading to lipids and proteins oxidation end products and tissue damages^2^. Antioxidants (SOD, CAT, GPX, and GSH) scavenged ROS. SOD catalyzes the dismutation of superoxide O_2_^⋅−^ into oxygen, and hydrogen peroxide, CAT catalyzes the breakdown of hydrogen peroxide (H_2_O_2_) to water (H_2_O) and oxygen (O_2_), GSH and GPX also play a similar role^25^. We supposed that antioxidants were oxidized by free radicals that reduced their activities in consequence of methionine supplementation.

Our results showed decline under condition of oxidative stress in the three 20S proteasomal peptidase activities (chymotrypsin-like, trypsin-like, caspase-like) in treated rats, accompanied by decrease of their associated subunits expression in order (β5, β2, β1) that indicated proteasome dysfunction. Similarly, many studies reported impaired proteasome targeted by damaged and ubiquitinated proteins in diseases during oxidative stress situation that decreased its 20S proteasomal activities and also β and α subunits expression^16,19,26^. In addition, accumulated oxidized proteins (PC) demonstrated by our findings are so in favor of inhibited proteasome observed. We believe, It is interesting to verify all proteasome subunits expression and also identify damaged proteins targeted these subunits. Moreover, evidence that 20S proteasome is less susceptible towards oxidative damage and responsible to eliminate oxidized proteins in an ATP-independent, and ubiquitin-independent, manner than the ATP-dependent 26S proteasome, known to degrade ubiquitinated substrates^16^. However, in severe OS, both 20S and 19S proteasomal subunits can be modified by extensive binding damaged proteins as    4hydroxynonenal-induced adducts (HNE), protein carbonyls (PC), products of *S*-glutathionylation (*S*-Glut.) or glycoxidation (Glyc) that inhibited proteasome^16,26^. Das et al.^19^ showed that Bortezomib-induced decline in proteasomal activity caused severe hepatocellular injury in human NAFLD patients in correlation of proteasomal genes and hepatocytes highly sensitive to oxidative stress.

As biomarkers of liver toxicity, methionine rich diet in rats elevated levels of hepatic enzymes (AST, ALT, LDH, and ALP) and bilirubin but diminished albumin. These alterations indicated loss in critical liver functions. Same reports^1,12,27^ revealed altered liver enzymes and proteins related to hepatotoxicity during methionine caused HHcy in animals and human. It is well established that injured hepatocytes dumped their proteins content in blood in cause of altered tissue structure^27^.

Histological examination of liver sections in high methionine rats showed marked alterations in the liver tissue, as inflammatory cells infiltration, ballooned hepathocytes, necrosis and sinusoid hemorrhage. These observations are similar to others^27,28^. Furthermore, altered liver structure confirmed the release of hepatic proteins in plasma as outlined above.

In term of inflammatory markers, methionine in treated rats, increased both C-reactive protein (CRP), the first indicator of inflammation in plasma, and liver cytokines (tumor necrosis factor-α and interleukin-6). These results are in accordance with previous reports^11,12^, that revealed higher parameters of inflammation related to hyperhomocysteinemia. Inflammation is a biological process of the immune system in response to damaged cells but excessive inflammation contributed to diseases^20^. Interestingly, inflammatory cell infiltration and necrosis previously observed in our histological findings, strongly confirmed these increased proinflammatory (CRP, cytokines (TNF-αand IL-6). Furthermore, it is well known that active proteasome degrades IκΒ inhibitory proteins and activates nuclear factor-kappa B (NF-κB) to enhance a variety of proinflammatory cytokines^17^. In contrast we showed inhibited proteasome in treated rats with occurrence of increased inflammation but it consistent with earlier study^20^. Proteasome mechanisms regulated inflammation requires more investigation in liver injury.

Recently several researches^1,19,22^ focused on methionine supplementation role in chronic liver diseases. Our study highlights the ability of high methionine diet feeding to induce intermediate hyperhomocysteinemia enhancing oxidative stress and leading to proteasome dyfunction and affecting hepatocytes.

Therefore, reducing high methionine intake in diet is important to avoid hepatotoxicity. Strategies recommended supplementation of folate, vit B12, vit B6 and cysteine to lower hyperhomocysteinemia^2,22^, as well as the use of natural and synthetic antioxidants to cope with ROS generation and protect against cell injuries^2,13,22^. Moreover, proteasome inhibitors have been recognized as a therapeutic target in diseases. Earlier research^20^ showed that proteasome inhibitors reduced liver damage and inflammation due to chronic ethanol feeding caused impaired proteasome and release of cytokines. However, we think that proteasome activators could have potential effects against proteotoxic liver injury by reducing oxidative stress. As mentioned in neurodegenerative diseases that enhancement of proteasome activity has much therapeutic potential but is still a relatively unexplored field^29^. Thus, challenges approaches towards proteasome mechanisms in liver diseases.

We conclude the harmful effect of high-methionine diet, as a hepatic risk factor that induced oxidative stress throughout hyperhomocysteinemia, leading to proteasome dysfunction, hepatotoxicity and inflammation. The toxic effect of methionine in liver disturbs functions and associated liver diseases which represent a major public health problem worldwide, to date without effective treatments. Further studies should be needed to elucidate mechanism of methionine pathophysiology to design in future possible new therapeutic measures.

## Methods

### Animal experimental protocol

All rats experiments were conducted in accordance with the Arrive guidelines (PLoS Bio8 (6), e1000412, 2010) (https://arriveguidelines.org/) and were approved by the Algerian National Council of Ethics for Health Sciences under the authority of Ministry of Health. Furthermore, the protocol was approved by the local ethical committee of Khenchela University in Algeria.

Male albino Wistar rats aged of 3 months old (n = 16) and weighing (200–240 g) were provided from Pasteur Institute (Ministry of Health) in Algiers, Algeria. Rats were kept in cages at controled room 12 h light/dark cycle, temperature (24 ± 1 °C) and humidity (70%) with free access to food and water. After two weeks of adaptation period, they were divided into two groups, controls and treated (n = 08/group). Treated rats received orally l-methionine (1 g/kg/day) for 3 weeks as previously described^30^, with modification, to create hyerhomocysteimic pathological rat model by referring every week to the measured total plasma homocysteine (tHcy) levels.

Rats were sacrificed by decapitation after overnight fasting. Plasma was separeted at 3000 rpm for 20 min at 4 °C and stored at − 80 °C for the biochemical analysis. Livers were homogenized, in ice-cold lysis buffer containing 30 mM Tris, pH 7.2, 1 mM dithiothreitol (DTT), 1% Triton X for 2 min with homogenizer (Janke-Kunkel, Inka-Labotecknik) and then centrifuged at 12,000 rpm for 15 min at 4 °C. Supernatants were taken for total protein determination by the method of Bradford using bovine serum albumin (BSA) as standard^31^ and were immediately frozen at − 80 °C for later assays. Liver specimens were fixed in 10% buffered formalin for the histological examination.

### Homocysteine analysis

Total homocysteine (tHcy) level was detected in plasma by an enzyme immunoassay using Axis Homocysteine EIA Kit (Catalog number AX51301) (IBL International, Germany). Hcy was expressed as μmol/l. The principle of the assay is based on the reduction of protein-bound homocysteine and its other conjugated forms (Prot-SS-Hcy, Hcy-SS-Hcy, *R1-SS-Hcy,*R1 is a residue thiol) to homocysteine free by dithiothreitol (DTT), which is subsequently enzymatically converted to S-adenosyl-l-homocysteine (SAH) by an SAH hydrolase in the presence of excess adenosine (AD). Following the Kit's instructions, sample reduction pretreatment is carried out by adding 500 μl of the previously prepared SPS (Sample Preparation Solution) (4.5 ml of reagent A, 0.25 ml of reagent B, 0.25 ml of reagent C) to 25 μl of calibrators, sample or control. After homogenization, the tubes covered by the parafilm are placed for 30 min at 37 °C. Before cooling the tubes, 500 μl of reagent D are added, mixed and incubated for 15 min at 18–25 °C. Then, 500 μL of reagent E are added, and incubated for 5 min at 18–25 °C. For the enzyme immunoassay, 25 μl of the calibrators, controls and treated samples are placed in the wells of the SAH-coated microtiter plates, and are immediately added to 200 μl of reagent F and incubated for 30 min at 18–25 °C. After washing 3 times with 400 μl of diluted washing buffer, 100 μl of reagent G are coupled and incubated for 20 min at 18–25 °C then washed with diluted washing buffer (3 × 400 μl). Then 100 µl of reagent H are placed in each well and incubated for 10 min at 18–25 °C. Likewise, 100 µl of reagent are also distributed in each well of the plate and are automatically balanced to ensure the mixing of the samples. Optical density is measured at 450 nm with an ELISA reader. The absorbance is inversely proportional to the total concentration of homocysteine in the plasma. The concentration of homocysteine is determined in the sample using a standard curve produced by the calibrator (S-adenosyl-l-homocysteine: 2.0–50.0 μmol/l).

### TOS/TAS and OS measurements

TOS (total oxidant status) and TAS (total antioxidant status) were assessed by colorimetric methods as described^32,33^. Results were respectively expressed as (μmol H_2_O_2_Eq/l) and (mmol Trolox Eq/l). OSI (oxidative stress index) was calculated as follows: OSI = (TOS/TAS) × 100.

### MDA determination

MDA (malondialdehyde) levels expressed as nmol/mg proteins were determined by a spectrophotometric method as previously described^34^. Thiobarbituric acid (TBA) reacts with malondialdehyde to form a pink complex which absorbs at 532 nm. The dosage is carried out on 100 μl of homogenate added to 0.6 ml of phosphoric acid (1M) and 0.2 ml of thiobarbituric acid (0.6%). After incubation in closed tubes in boiling water bath at 95 °C for 45 min, then placed in an ice bath for 10 min. 0.8 ml of n-butanol are added to this mixture, then centrifuged at 2000 rpm for 10 min. The MDA contents are quantified by reading the difference in absorbance of the pink-colored sample at 520 and 535 nm against the reactive white and by referring to a standard MDA curve prepared by hydrolysis of 1.1.3.3. tetrahydroxypropane.

### PC determination

PC (protein carbonyls) levels expressed as nmol PC/mg proteins, were measured using an Oxi Select™ Protein Carbonyl Spectrophotometric Assay Kit (Catalog number STA-315) (Cell Biolabs, Inc). The principle of the assay is based on the measurement of hydrazone derivatives of proteins, using 2, 4 dinitrophenyl hydrazine (DNPH), a classic reagent used with proteins. Following the Kit instructions, 125 µl of homogenate (1–10 mg/ml) is incubated with 0.5 ml of DNPH (2 mg/ml) for 45 min in the dark and with occasional shaking. For each homogenate, the sample blank (without DNPH) is prepared by the addition of DNPH diluent. Then, 0.625 ml of trichloroacetic acid (TCA) is added and after 10 min of incubation on ice, the whole is centrifuged at 10,000×g for 10 min at 4 °C. The supernatant is removed and the pellet is washed 5 times with 0.5 ml of ethanol-ethyl acetate (1:1, v/v). After the last wash, the pellet is suspended in 125 μl of protein solubilization solution and incubated for 10 min at 37 °C, then centrifuged for 10 min at 10,000×g to remove insoluble debris. Protein concentrations are estimated for each sample. Then, the supernatant is transferred to a 1cm mini tank and the absorbance is read against the blank for each sample on the spectrophotometer at 375 nm. Using the molar absorption coefficient of DNPH at 375 nm, (ε) equivalent to 22,000 M^−1^ cm^−1^, carbonylated proteins are measured and are expressed in nmol/mg proteins), by the equation: [Protein Carbonyl (M) = A375 nm/22000 M^−1^] = [Protein Carbonyl (nmol/mg) = A 375 nmx 45.45 (nmol/mg) = Protein Carbonyl (nmol/mg)/Proteins.

### Non-enzymatic and enzymatic antioxidants measurements

Reduced glutathione (GSH) content was assayed using a colorimetric technique as previously described^35^ based on the measurement of the optical absorbance at 412 nm of DTNB (2-nitro-5-mercapturic acid which results from the reduction of 5,5 dithio-bis-2-nitrobenzoic acid) by the groups (-SH) glutathione. GSH content was expressed as nmol/mg proteins. Catalase (CAT) activity was measured as previously reported^36^ and was expressed as (U/mg proteins). Superoxide dismutase (SOD) activity was carried out as previously described^37^ by using the inhibition of autoxidation of pyrogallol and was expressed as (U/mg proteins). Glutathione peroxidase activity (GSH-Px) expressed as (μmol/mg proteins) was assayed by the method^38^ based on the reaction between glutathione remaining and DTNB (5, 5-Dithio-bis (2-nitrobenzoic acid) to form a complex that absorbs maximally at 412 nm.

### Proteasome activities assay

Proteasome activities of 20S core: CT-L (chymotrypsin-like), T-L (trypsin-like), C-L (caspase-like) were assayed in the supernatants (20 µM) using specific fluorogenic substrate (70 µ M ) for each activity respectively Suc-LLVY-AMC (Calbiochem,USA), Boc-LSTR-AMC (Sigma Aldrich,France) and Z-LLE-AMC (Sigma Aldrich, France) and calculated as the difference between the presence or the absence of specific proteasome inhibitor MG132 (Z-Leu-Leu-Leu-al (Sigma Aldrich,France) (10 µM) for 1 h at 37 °C as previously described^39^. Activities were expressed as relative fluorescent units (RFU)/60 min/mg proteins with (355 excitation/460 emission) by microtiter plate fluorometer Mithras LB940 (Berthold Technologies, Bad Wilbad, Germany**) .**

### Western blot analysis of 20S βproteasome subunits expression

Proteins (25 μg), after were supplemented with both 1% proteases inhibitors cocktail and phosphatases inhibitors,were then separated on 4–12% on sodium dodecyl sulfate polyacrylamide gel (Invitrogen, Cergy-Pontoise, France) and transferred to a nitrocellulose membrane (GE Healthcare, Orsay, France) for 1 h at 100 V as previously described^40^. The membrane was blocked for 1 h at room temperature with 5% (w: v) non-fat dry milk in Tris-buffered saline (TBS, 10 mmol/l, Tris, pH 8; 150 m mol/l NaCl) plus 0.1% Tween 20) followed by overnight incubation at 4°C with specific primary antibodies, mouse anti (20S subunit β1, 20S subunit β2) (1:1000, Biomol) or mouse anti β-actin (1:1000, Sigma Aldrich), rabbit anti β5 (1:1000, Biomol). After three washes in TBS/0.1% Tween 20, at 1 h incubation with secondary antibodies, horseradish peroxidase-conjugated goat anti rabbit or anti mouse IgG (1:5000, Santa Cruz Biotechnology) was performed, followed by three additional washes, immunoreactive proteins were revealed by using Enhanced Chemiluminescence (ECL) detection system (GE Healthcare). Protein bands were quantified by densimetry using Image scanner III and Image Quant TL software (GE Healthcare).

### Plasma biochemical analysis

AST (aspartate aminotransferase), ALT (alanine aminotransferase), LDH (lactate dehydrogenase), ALP (alkaline phosphatase), TBil (total bilirubin) and Alb (albumin) were assayed spectrophotometrically with an auto-analyzer in accordance with the package inserts of commercial kits respectively (Ref. 1001160, Ref. 1001170, Ref. 1001260, Ref. 1001130, Ref: 1001046, Ref. 1001020) (Spin react diagnostic kits, Spain). CRP levels expressed in mg/L were measured by Rat C-Reactive Protein (CRP) ELISA Kit (Catalog number: 88-7501) (Invitrogen, USA).

### Liver histological examination

Liver specimens fixed in 10% buffered formalin, were dehydrated and embedded in paraffin. Liver longitudinal sections of 4 μm thick were then stained with hematoxylin–eosine (H–E) for light microscopic observation^41^.

### TNF- and IL-6 assays

TNF- and IL-6 levels were determined using sandwich Enzyme-Linked Immunosorbent Assay (ELISA) respectively by (Rat TNF-alpha ELISA Kit (ERA56RB), and Rat IL-6 ELISA Kit (ERA31RB), Invitrogen, USA) following the manufacturer's instructions. Results were expressed as pg/mg proteins.

### Statistical analysis

Data were compared by Mann–Whitney test using Graph Pad Prism 5.0 (Graph Pad software Inc. San Diego.CA, USA) and were expressed as mean ± SEM. For all results, P values < 0.05 were considered statistically significant.

### Supplementary Information

Supplementary Figures.

## Data Availability

The data that supported the findings of this study are available from the corresponding author FD upon request.
