# Supplementary material for: High methionine diet mediated oxidative stress and proteasome impairment causes toxicity in liver
Source: Sci Rep. 2024 Mar 6;14:5555. doi: 10.1038/s41598-024-55857-1 (PMC10917754; doi:10.1038/s41598-024-55857-1)
Supplement: Supplementary file 1 — Supplementary Figures. [file 41598_2024_55857_MOESM1_ESM.pdf]

High methionine diet mediated oxidative stress and proteasome impairment causes toxicity in liver.

Faouzia Derouiche [1,2,*], Randa Djemil [1,2], Fatima Zohra Sebihi [2], Lilia Douaouya[2], Maamar Hichem[2], Katia Benjemana [1,2]

[1] Biotechnology, Water, Environment and Health Laboratory, Faculty of Natural and Life Sciences, University Abbes Lagherour Khenchela., Algeria

[2] Department of Molecular and Cellular Biology, Faculty of Natural and Life Sciences, University Abbes Lagherour Khenchela., Algeria.

Supplementary Figure 1

Homocysteine protocle

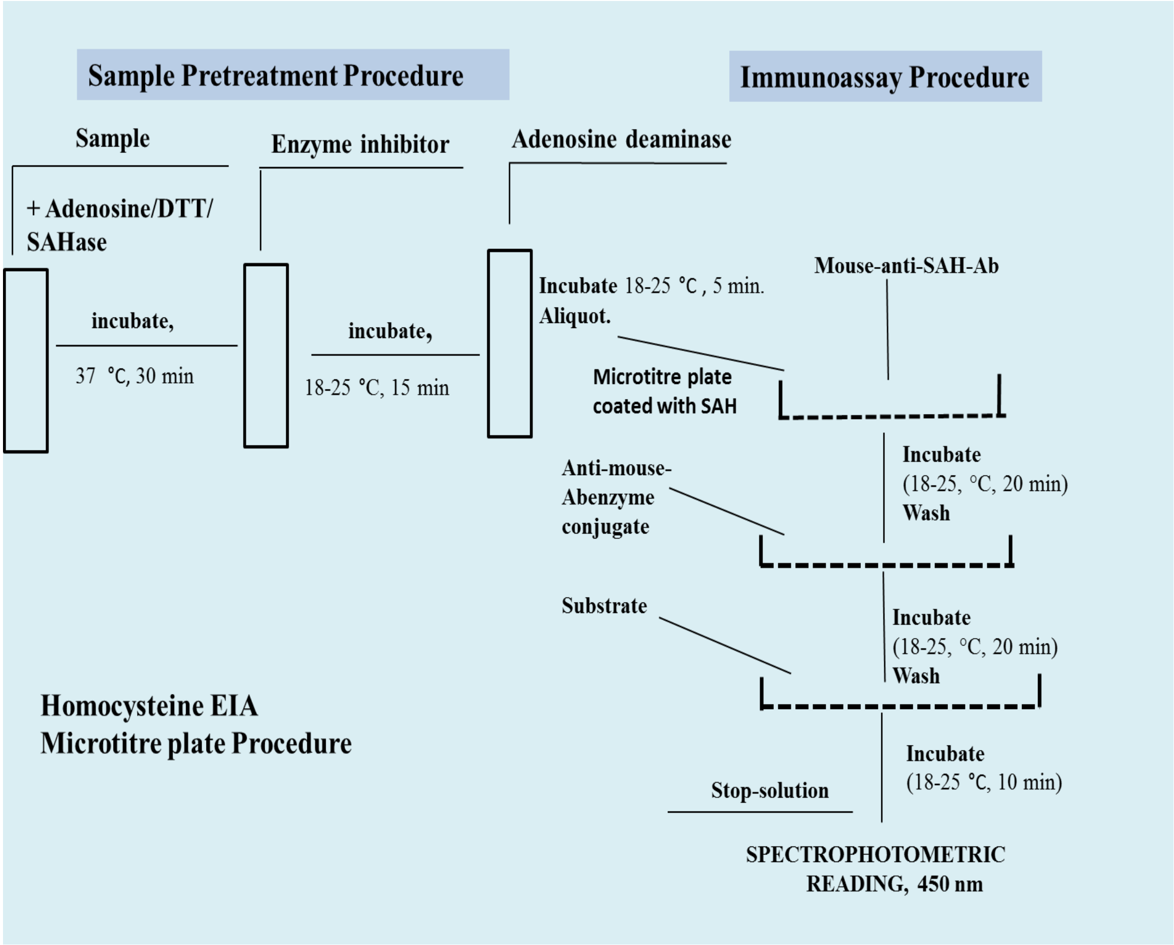

Supplementary Figure 2

Effect of L-methionine supplementation on 20S proteasome activities

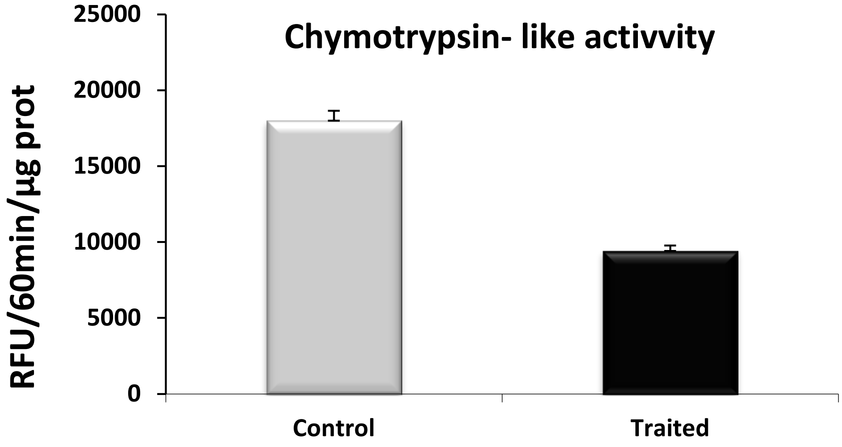

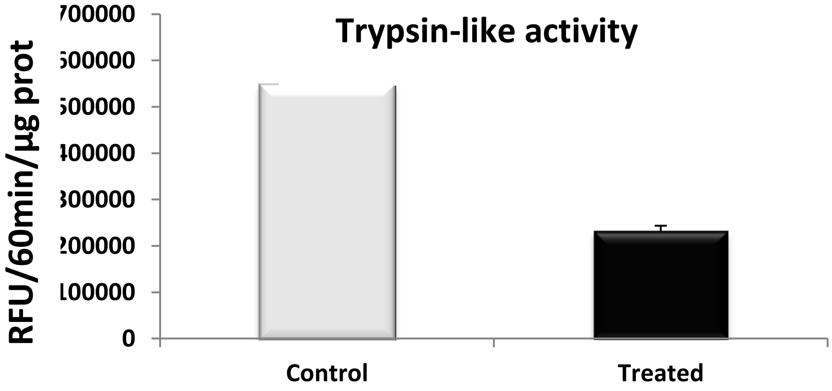

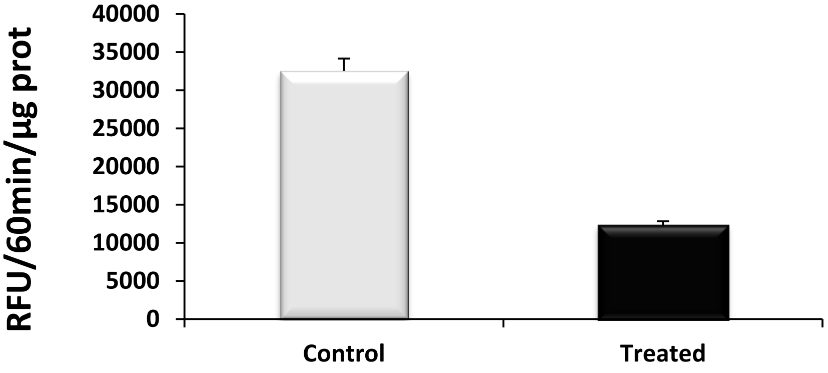

Caspase –like activity

Supplementary Figure 3

Western blots of 20S β subunits

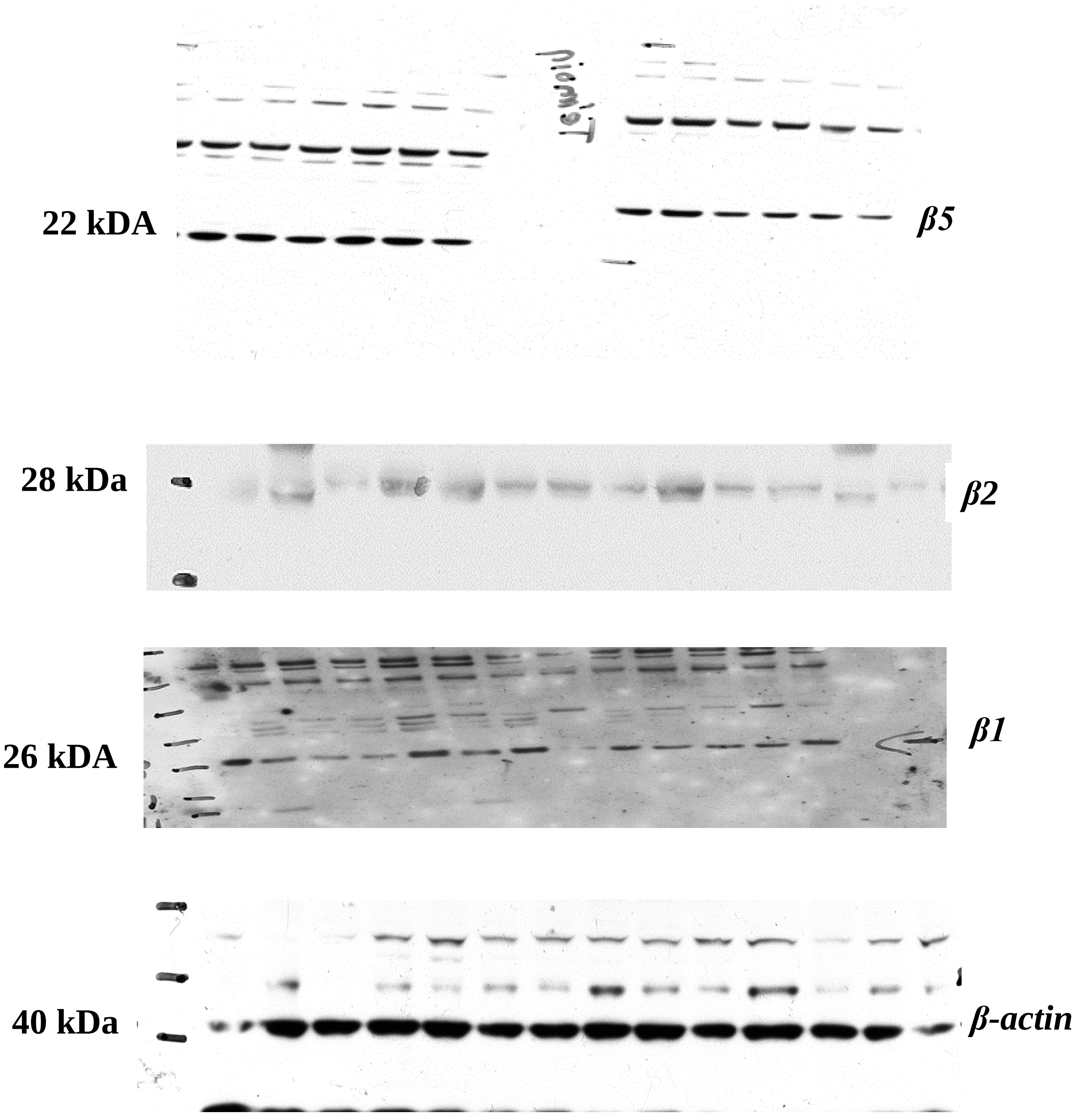

Supplementary Table 1

Table / proteasome peptidase activity measurement on microplaque									
PSIII = Suc-LLVY: Substrat for chymotrypsin-like activity					Volume (µl)				
Samples	[ ] pro(µg/ µl)	25µg	Conditions	Plaque Position	Tampon activity	Proteins	DMSO	PSIII	MG 132
None				1A	0,0	0,0	0	0	0
Tris HCl				1B	100,0	0,0	0	0	0
DMSO				1C	90,0	0,0	10	0	0
				1D	90,0	0,0	10	0	0
PSIII				1E	95,0	0,0	0	5	0
				1F	95,0	0,0	0	5	0
MG 132				1G	95,0	0,0	0	0	5
				1H					
1<br>2	39,62<br>30,92	0,63<br>0,81	PSIII	2A	89,4	0,6	5	5	0
			MG 132	2B	89,4	0,6	0	5	5
			PSIII	2C	89,2	0,8	5	5	0
			MG132	2D	89,2	0,8	0	5	5
